# Dynamic dissipative control for fuzzy distributed parameter cyber physical system under input quantization and DoS attack

**DOI:** 10.1371/journal.pone.0311215

**Published:** 2024-10-03

**Authors:** Jingzhao Chen, Liming Ding, Tengfei Li

**Affiliations:** 1 School of Electronic Information and Intelligent Manufacturing, SIAS University, Zhengzhou, Henan, China; 2 School of Computer and Artificial Intelligence (School of Software), Huaihua University, Huaihua, Hunan, China; 3 School of Mechatronic Engineering and Automation, Foshan University, Foshan, Guangdong, China; Xi’an Jiaotong University, CHINA

## Abstract

This article explores the dissipative control for a class of nonlinear DP-CPS (distributed parameter cyber physical system) within a finite-time interval. By utilizing a Takagi-Sugeno (T-S) fuzzy model to represent the system’s nonlinear aspects, the studied system is formulated as a class of fuzzy parabolic partial differential equation (PDE). In order to optimize network resources, both the system state and input signal are subjected to quantization using dynamic quantizers. Subsequently, a dynamic state control strategy is proposed, taking into account potential DoS attack. The finite-time boundedness of the fuzzy parabolic PDE is analyzed, with respect to the influence of quantization, through the construction of an appropriate Lyapunov functional. The article then presents the conditions for finite-time dissipative control design, alongside the adjustment parameters for the dynamic quantizers within the fuzzy closed-loop system. Furthermore, the decoupling of interlinked nonlinear terms in the control design conditions is achieved by using an arbitrary matrix. Finally, an example is provided and the simulation results indicate the effectiveness of the dissipative control method proposed.

## Introduction

Benefiting from the development of computer science, networking communication, and physical devices, cyber physical systems (CPS) have witnessed rapid development in the past century. CPS play a key enabler to connect the physical layer, computing layer, and network layer [1]. As predicted in [2], the following generation of industry will be constructed based on CPS. The performances of many industrial systems relay on spatial variables as well as time variables inherently, the ordinary diffrential equation described systems and the finite-dimensional time-dependent systems are not suitable to model these systems [3]. This kind of CPS which contain diffusion, can be frequently characterized with distributed parameter systems [4]. The control design for a system is a practical issue and has been widely studied, such as, the adaptive control [5], the neural network tracking control [6], and the synchronization controller design [7]. And stability analiysis [8, 9] which is also an important issue for the control systems have also been proposed. The last century has witnessed the rapid development research of distributed parameter cyber physical systems (DP-CPS), some stability analysis and control design methods [10, 11] are also been provided. However, because of the intricate and nonlinear nature of system architectures, the interdependence of uncertain control parameters, and the presence of both external and internal disturbances, the controller design for DP-CPS still faces a multitude of challenges.

To establish communication between the physical layer and computing layer, CPS data is transmitted over the network. This offers the benefits of leveraging the flexibility of the physical layer while reducing installation and budget requirements. Within the network layer of the CPS, a significant characteristic is the volume of data. However, due to the limited bandwidth of the network, this results in constraints on the communication channel’s capacity. If the data of CPS that needs to be transmitted is to much, the network congestion which may results in instability of the system or failure of the control target will occur [12]. In this scenario, signal quantization before transmission is considered as an effective approach to address this issue and is extensively utilized in networked control systems. However, it’s important to note that quantization errors can arise alongside signal quantization, potentially hindering the attainment of control objectives and impacting control performance, as highlighted in [13]. Thus, the research on controller design for DP-CPS with quantization is necessary and pregnant. Although there are some results on the quantized control for DP-CPS have been reported (see [14, 15] and the references therein), these achievements are mainly based on the kind of static quantizer. Static quantizers are characterized by time invariance and are defined by memoryless nonlinear functions, making their construction straightforward. To satisfy the prescribed performance of the system, unlimited quantization levels of the static quantizer are necessary. It’s crucial to note that the system’s performance cannot be assured when dealing with a static quantizer limited by a restricted quantization level. In this scenario, dynamic quantizer, which can dynamically adjust the quantizer parameters to obtain different levels of quantization ranges and quantization errors, can be applied to solve this problem. In this research, the dynamic quantizer proposed in [16] is used to simply the quantization rules.

While the deep integration of computing, physical plants, and communication networks offers numerous advantages for CPS, the openness of communication networks also poses information security risks that can easily result in production safety issues in physical systems [17]. Particularly, control terminals frequently encounter the threat of network attacks during data transmission. The network attack is one of the research issues in security problems. Undoubtedly, the network attack constitute a significant factor contributing to the destabilization or even complete failure of CPS. According to the form of attacks, the research in the existing works maninly focuses on three types of attacks: false data injection, denial of service (DoS), and replay attack [18]. With the consideration of attack, plenty of results have been presented, such as, security control [19] filtering design [20], and secure synchronization [21]. For the DP-CPS with attack, event-triggered security control strategy with pointwise measurements is studied based on sampled-data technique [22]. After that, leader-following consensus control for multiagent systems modeled with parabolic partial differential equations is reported in [23]. More recently, the pinning synchronization sampled-data control method for reaction-diffusion neural networks by establishing a directed neural network model to deal with the random deception attack [24]. More recently results can be referred in [25]. Although there are already some results have been achieved, these results have not considered the signal quantization of DP-CPS. The quantized control under DoS attack for DP-CPS needs further study.

Due to the complexity of the system, most practical system models are essentially nonlinear, which leads to the fruitful achievements of linear system can not be be applied to nonlinear DP-DPS directly. To deal with the nonlinearities of the industrial systems, many useful methods have been developed, such as, Takagi-Sugeno (T-S) fuzzy model [11–15, 25–27], type-2 fuzzy model [28], Lipschitz function with a Lipschitz constant [29]. In this study, a T-S fuzzy model is picked up to represent the nonlinear terms of DP-CPS. By using the T-S fuzzy models, many results of the control design methods are proposed [11, 14, 15, 25]. The T-S fuzzy model provides an accurate approximation method to the original system model, and its simple fuzzy rules and solving methods greatly facilitate the control design. Inspired by the excellent results, the dynamic control strategy with input quantization under DoS attack will be studied for DP-CPS by employ the T-S fuzzy model.

During the process of system control design, requirements related to control performance are often encountered. Currently, the most widely utilized performance index is the dissipative performance. The extended dissipative performance is regarded to be more general than $H_{\infty }$ performance [30], *l*_2_-*l*_∞_ performance [31], and passivity performance [32]. It is imperative to ensure control performance within a finite time interval. Several significant research findings have been documented, such as the investigation of observer-based dissipative control performance for DP-CPS over a finite-time interval, as outlined in [33]. Additionally, the exploration of a fuzzy dissipative control strategy considering time-varying delay and incomplete transition probabilities for a specific group of Markovian jump DP-CPS has also been presented in the literature [34]. However, there is limited research on solving the finite-time dissipative boundedness problem for DP-CPS with quantization and DoS attack. Thus, the finite-time dissipative control of DP-CPS needs further investigation and is of important practical significance.

Motivated by the existing achievements, the aim of this article is to investigate finite-time dissipative boundedness of fuzzy DP-CPS modeled with the parabolic partial differential equation (PDE) via dynamic control strategy with input quantization and DoS attack. This study offers sufficient criteria for the determined control gains and dynamic quantizer parameters, achieved through the application of specific decoupling techniques. In summary, the contributions can be outlined as follows:

1) The state and control input signals are subject to quantization using dynamic quantizers. These quantizers streamline the quantization rules in contrast to static ones, resulting in resource savings and mitigating network congestion.
2) The DoS attack which is represented as the Bernoulli stochastic process is considered, and then the spatial-independent dynamic controller is designed for the nonlinear DP-CPS.
3) The criteria with the finite-time dissipative boundedness are established by formulating Lyapunov functionals. Additionally, introducing a method for determining, rather than solving, some process matrices. This method has the advantage of mitigating the conservative constraints arising in the controller design.

The structure of the subsequent sections is as follows: Section “Problem Statements” deals with the problem formulation and provides preliminary information. Section “Controller Structure” introduces the spatial-independent dynamic state controller with quantization under DoS attack. section “Controller Design Condition and Stability Analysis” and presents the control criteria and the analysis of the stability. In section “Simulation Studies”, to demonstrate the feasibility and efficiency of the designed strategy, a numerical simulation is provided. Finally, section “Conclusion” offers concluding remarks for this article.

### Notations

The symbols R, $R^{+}$, $R^{n}$, and $R^{m\times n}$ correspond to the sets of real numbers, positive real numbers, *n*-dimensional Euclidean space, and real matrices with dimensions *m* × *n*, respectively. The transpose of the matrix *X* is represented as *X*^*T*^. The Hilbert space $H^{n}≜L_{2}\left(\left[0,l\right];R^{n}\right)$ has the properties: The inner product between *g*_1_(⋅) and *g*_2_(⋅) is given by $\langle g_{1}\left(\cdot \right),g_{2}\left(\cdot \right)\rangle =\int_{0}^{l}g_{1}^{T}\left(x\right)\cdot g_{2}\left(x\right)dx$, and the norm of *g*_1_(⋅) is defined as $|g_{1}{\left(\cdot \right)|}_{2}=\sqrt{\langle g_{1}\left(\cdot \right),g_{1}\left(\cdot \right)\rangle }$. Here, $g\left(x\right):\left[0,l\right]\to R^{n}$ is a vector function that is square integrable, and *g*_1_(*x*), *g*_2_(*x*) are both elements of $H^{n}$. The function $h\left(x\right):\left[0,l\right]\to R^{n}$ is an absolutely continuous vector function with $d^{\bar{n}}h\left(x\right)/dx^{\bar{n}}$ of order $\bar{n}\geq 1$. Its norm is defined as ${\left|h\left(\cdot \right)\right|}_{W^{1,\bar{n}}}=\sqrt{\int_{0}^{l}\sum_{i=0}^{\bar{n}}\frac{dh^{T}\left(x\right)}{dx}\cdot \frac{dh(x)}{dx}dx}$. $W^{1,\bar{n}}\left(\left[0,l\right];R^{n}\right)$ designates a Sobolev space. The symbol *X*^−1^ denotes the inverse of the matrix *X*. The notation * indicates the symmetric part of a matrix, i.e., $\left[\begin{array}{cc} X_{1} & X_{2}\\ {}* & X_{3} \end{array}]=[\begin{array}{cc} X_{1} & X_{2}\\ X_{2}^{T} & X_{3} \end{array}\right]$, [*X* + *] = [*X* + *X*^*T*^]. The terms λ_*min*_(*X*) and λ_*max*_(*X*) represent the minimum and maximum eigenvalues of *X*. *col*{*X*_1_, *X*_2_} signifies $=[\begin{array}{cc} X_{1}^{T} & X_{2}^{T} \end{array}{]}^{T}$. The expressions $\frac{\partial X(s,t)}{\partial s}=X_{s}\left(s,t\right)$ and $\frac{\partial^{2}X\left(s,t\right)}{\partial s^{2}}=X_{ss}\left(s,t\right)$ denote the first-order and second-order partial derivatives of *X*(*s*, *t*) with respect to the variable *s*, respectively.

## Problem statements

This section introduces the studied DP-CPS along with some essential lemmas. Usually, the mathematical representation of a nonlinear distributed parameter CPS can take the form of the following parabolic partial differential equation PDE:

$$\begin{array}{c} \left\{ \begin{array}{cc} & z_{t}\left(s,t\right)=Az_{ss}\left(s,t\right)+f\left(z\left(s,t\right),s\right)+B\left(s\right)u\left(s,t\right)+C\left(s\right)\omega \left(t\right),\\ & y(t)=Dx(0,t), \end{array}\right. \end{array}$$ (1)

where *s* and *t* represent the positional value in space and time, respectively. *z*(⋅, *t*) denotes a state vector, *f*(*z*(*s*, *t*)) is a nonlinear function, *u*(*t*) is the control input, *y*(*t*) denotes the measurement output, *ω*(*t*) is the disturbance, A is a known positive definite symmetric matrix, *z*_*ss*_(*s*, *t*) is the second-order partial derivatives of *z*(*s*, *t*) with respect to the variable *s*, and *B*(*s*), *C*(*s*), and *D* are constant matrices.

The following Neumann boundary conditions for (1) is considered:

$$\begin{array}{c} \begin{array}{c} \frac{\partial z(s,t)}{\partial s}{{|}_{s=0}=\frac{\partial z(s,t)}{\partial s}|}_{s=l}=0,\;t>0. \end{array} \end{array}$$ (2)

According to [12], the T-S fuzzy model described in Eq (1) is presented as:

Plant rule *i*: **IF** $\varkappa_{1}\left(s,t\right)$ is *F*_*i*1_, …, and $\varkappa_{\bar{q}}\left(s,t\right)$ is $F_{i\bar{q}}$, **THEN**

$$z_{t}\left(s,t\right)=Az_{ss}\left(s,t\right)+A_{i}z\left(s,t\right)+B_{i}u\left(t\right)+C_{i}\omega \left(t\right),$$ (3)

Here, the premise variables are represented as $\varkappa \left(s,t\right)=[\varkappa_{1}\left(s,t\right),\varkappa_{2}\left(s,t\right),\ldots ,\varkappa_{\bar{q}}\left(s,t\right)]$, where $\bar{q}$ denotes the number of nonlinear elements. The parameter *ν* belongs to the set $1,2,\ldots ,\bar{q}$ and signifies the fuzzy sets. Additionally, $\kappa =2^{\bar{q}}$ denotes the number of fuzzy rules (denoted by **If-Then**), where the n-dimensional square matrices *A*_*i*_ signifie known constant ones with *i* being a member of a set S=1,…,κ.

The entire T-S fuzzy model for system (1) is derived:

$$\begin{array}{c} \left\{ \begin{array}{cc} & z_{t}\left(s,t\right)=Az_{ss}\left(s,t\right)+\sum_{i=1}^{\kappa }h_{i}\left(\varkappa \left(s,t\right)\right)\left[A_{i}z\left(s,t\right)+B_{i}u\left(s,t\right)+C_{i}\omega \left(t\right)\right],\\ & y(t)=Dz(0,t), \end{array}\right. \end{array}$$ (4)

*F*_*iυ*_(*ϰ*_*υ*_(*s*, *t*)) indicates the membership grade of *ϰ*_*υ*_(*s*, *t*) in *F*_*iυ*_, and the membership function of *ϰ*(*s*, *t*) in *F*_*iυ*_ complies with the following [35]:

$$\begin{array}{c} \begin{array}{cc} & \chi_{i}\left(\varkappa \left(s,t\right)\right)=\Pi_{\upsilon =1}^{l}F_{i\upsilon }\left(\varkappa_{\upsilon }\left(s,t\right)\right),\\ & h_{i}\left(\varkappa \left(s,t\right)\right)=\chi_{i}\left(\varkappa \left(s,t\right)\right)/\sum_{i=1}^{r}\chi_{i}\left(\varkappa \left(s,t\right)\right),\\ & \sum_{i=1}^{\kappa }h_{i}\left(\varkappa \left(s,t\right)\right)=1,h_{i}\left(\varkappa \left(s,t\right)\right)\geq 0,i\in S, \end{array} \end{array}$$ (5)

Select the quadratic energy supply function associated with (1) as:

$$J(t)=y^{T}\left(t\right)J_{1}y\left(t\right)+2y^{T}\left(t\right)J_{2}\omega \left(t\right)+\omega^{T}\left(t\right)J_{3}\omega \left(t\right).$$ (6)

To simplify, *h*_*i*_(*ϰ*(*s*, *t*)) is simplified with *h*_*i*_ throughout the remainder of this article.

We introducing the following four lemmas and two definitions in order to smoothly derive the results of this paper in the next section.

**Lemma 1**. [35] Assume the presence of a matrix $G\in R^{n\times n}$, where all elements are greater than zero (0<G), a positive scalar *l*, and a vector function *x*(⋅) belonging to the space $W^{1,2}\left(\left[0,l\right]\right)$. Under these conditions, the following can be deduced:

$$\begin{array}{c} \frac{1}{l}[\int_{0}^{l}x\left(s\right)ds{]}^{T}G[\int_{0}^{l}x\left(s\right)ds]\leq \int_{0}^{l}x^{T}\left(s\right)Gx\left(s\right)ds. \end{array}$$ (7)

**Lemma 2**. [12] If the inequality A+[BC+*]<0 holds true, with A, B, and C having appropriate dimensions, then, for any given scalar *ε*, there exists a matrix M such that:

$$\begin{array}{c} \begin{array}{c} {}[\begin{array}{cc} A & *\\ \varepsilon B^{T}+MC & -\varepsilon [M+*] \end{array}]<0. \end{array} \end{array}$$ (8)

**Lemma 3**. [12] If there exists a proper matrix G such that $0<G\in R^{n\times n}$, the inequality

$$\begin{array}{c} -2X^{T}Y\leq X^{T}G^{-1}X+Y^{T}GY, \end{array}$$ (9)

holds, where X and Y are matrixes (or scalars) with appropriate dimensions.

**Lemma 4**. [35] If there exists a well-defined matrix $0<O\in R^{n\times n}$ and a differentiable vector function *x*(⋅) with *x*(0) = 0 or *x*(*l*) = 0, then:

$$\begin{array}{c} \int_{0}^{l}{\dot{x}}^{T}\left(s\right)O\dot{x}\left(s\right)ds\geq \frac{\pi^{2}}{4l^{2}}\int_{0}^{l}x^{T}\left(s\right)Ox\left(s\right)ds. \end{array}$$ (10)

**Definition 1**. [Finite-Time Boundedness (FTB)] Assuming the presence of a positive definite matrix *R* and three positive constants *c*_1_, *c*_2_, and T, where *c*_1_ < *c*_2_, if there exists a controller *u*(*t*) for system (4) such that, for all *t* within the interval [0,T], the following conditions hold:

$$\begin{array}{c} \begin{array}{cc} & \underset{t_{0}\in \left[-\tau ,0\right]}{\text{sup}}\left\{\tilde{z}\left(t_{0}\right),\tilde{\phi }\left(t_{0}\right)\right\}<c_{1}\\ & \Rightarrow \int_{\Omega }z^{T}\left(s,t\right)Rz\left(s,t\right)ds<c_{2},t\in \left[0,T\right], \end{array} \end{array}$$ (11)

where $\tilde{z}\left(t_{0}\right)=\int_{\Omega }z^{T}\left(s,t_{0}\right)Rz\left(s,t_{0}\right)ds$, $\tilde{\phi }\left(t_{0}\right)=\int_{\Omega }\phi^{T}\left(s,t_{0}\right)R\phi \left(s,t_{0}\right)ds$. Then the system state of (4) under the control *u*(*t*) is said to be finite-time boundness with respect to $\left(c_{1},c_{2},T\right)$.

**Definition 2**. [32] [Extended Dissipative Finite-Time Boundedness (EDFTB)] Given *α* > 0 and *J*_1_, *J*_2_, and *J*_3_, where both *J*_1_ and *J*_3_ are symmetric matrices, if system (1) conforms to Definition 1 and there exists a constant $\hat{\alpha }$ that satisfies the following condition:

$$\begin{array}{c} \begin{array}{c} \int_{0}^{T}J\left(t\right)dt\geq \alpha \int_{0}^{T}\omega^{T}\left(t\right)\omega \left(t\right)dt+\hat{\alpha }. \end{array} \end{array}$$ (12)

then the system with the function *J*(*t*) exhibits extended dissipative finite-time boundedness concerning the parameters (*c*_1_, *c*_2_, T, *α*, *J*_1_, *J*_2_, *J*_3_). The dissipativity performance bound is *α*.

The definition of the quantizer *q*(*t*) from [36] is applied in this context, with *H*_*q*_ > 0 and Δ_*q*_ > 0 representing the range and error bound, provided that:

$$\begin{array}{c} \begin{array}{cc} & \left\|q\left(t\right)-t\right\|\leq \Delta_{q},if\left\|t\right\|\leq H_{q},\\ & \|q\left(t\right)\|>H_{q}-\Delta_{q},if\left\|t\right\|>H_{q}, \end{array} \end{array}$$ (13)

And the dynamic quantizer presented in [36] is adopted:

$$\begin{array}{c} \begin{array}{cc} & q_{\mu }\left(t\right)=\mu q\left(\frac{t}{\mu }\right), \end{array} \end{array}$$ (14)

where *μ* is the dynamic parameter to be determined in the control design.

## Controller structure

In this section, it’s worth noting that actuators may experience the attack in practical engineering scenarios. As a result, a dynamic state feedback controller for the parabolic PDE system (1) that incorporates quantization under DoS attack will be introduced. The quantized dynamic state feedback controller under DoS attack is considered as:

$$\begin{array}{c} \left\{ \begin{array}{cc} & \phi_{t}\left(s,t\right)=A_{d}\phi \left(s,t\right)+B_{d}q_{\mu }(x(s,t))\\ & u\left(t\right)=F\left(t\right)q_{\mu }[\int_{0}^{l}C_{d}\phi \left(s,t\right)ds], \end{array}\right. \end{array}$$ (15)

involving the undetermined gain matrices *A*_*d*_, *B*_*d*_, and *C*_*d*_, where *ϕ*(*s*, *t*) represents the state of the dynamic controller, the initial values *ϕ*(*s*, 0) = *ϕ*_0_(*s*), and *F*(*t*) = *diag*{*f*_*c*1_(*t*), …, *f*_*ci*_(*t*), …, *f*_*cm*_(*t*)} is the attack decided by a Bernoulli stochastic process:

$$\begin{array}{c} \begin{array}{c} Prob\left\{f_{ci}\left(t\right)=1\right\}={\bar{f}}_{ci},Prob\left\{f_{ci}\left(t\right)=0\right\}=1-{\bar{f}}_{ci}, \end{array} \end{array}$$ (16)

where *f*_*ci*_(*t*) ∈ [0, 1] represents the potential failures of the *i*th actuator, while $\bar{f}ci\in \left[0,1\right]$ is a predetermined constant. Let $\bar{F}$ be defined as $\bar{F}=\text{diag}\;\bar{f}c1,\ldots ,\bar{f}ci,\ldots ,\bar{f}cm$. Then:

$$\begin{array}{c} \begin{array}{c} E\left\{F\left(t\right)-\bar{F}\right\}=0,E\left\{F\left(t\right)\right\}=\bar{F}. \end{array} \end{array}$$ (17)

Subsequently, employing the quantizer’s definition outlined in (13) and (14), the aforementioned controller can be expressed as:

$$\begin{array}{c} \left\{ \begin{array}{cc} & \phi_{t}\left(s,t\right)=A_{d}\phi \left(s,t\right)+B_{d}z\left(s,t\right)+B_{d}\bar{\vartheta }\left(s,t\right),\\ & u\left(t\right)=F\left(t\right)[\int_{0}^{l}C_{d}\phi \left(s,t\right)ds+\hat{\vartheta }\left(t\right)], \end{array}\right. \end{array}$$ (18)

where $\bar{\vartheta }\left(s,t\right)=\bar{\mu }\left(s,t\right)[q(\frac{z(s,t)}{\bar{\mu }\left(s,t\right)})-\frac{z(s,t)}{\bar{\mu }\left(s,t\right)}]$ and $\hat{\vartheta }\left(t\right)=\hat{\mu }\left(t\right)[q(\frac{\int_{0}^{l}C_{d}\phi \left(s,t\right)ds}{\hat{\mu }\left(t\right)})-\frac{\int_{0}^{l}C_{d}\phi \left(s,t\right)ds}{\hat{\mu }\left(t\right)}]$.

By substituting the controller into (1), we obtain the derived quantized state feedback control system and then the following results can be obtained.

**Fig 1:**
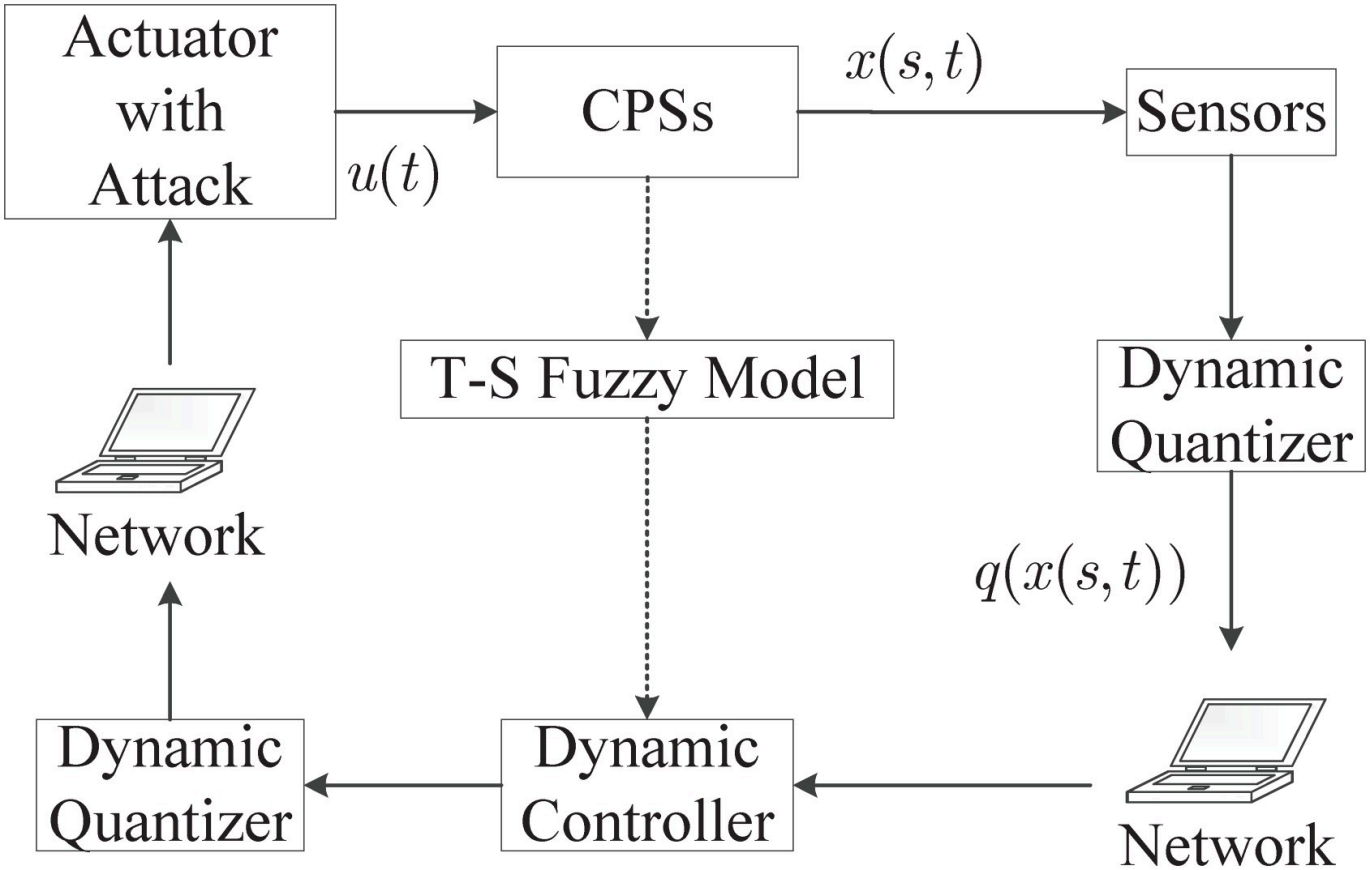
Framework of the control strategy.

Fig 1 describes the mechanism of the control strategy.

**Remark 1**. Compared with the controller presented in [17], a kind of spatial-independent dynamic controller (15) is designed based on the quantized state and control input signals. And this kind of dynamic controller can overcome the limitation that the controller presented in [17] can not stabilize any systems with an odd number of real eigenvalues greater than unity.

## Controller design condition and stability analysis

**Theorem 1**. Consider the closed-loop system (4), if there exist positive matrices *P*_1_ and *P*_2_, proper dimensional parameters *P*_2*A*_, *P*_2*B*_, *V*, *U*, scalars *ρ*_*u*_, *ρ*_*y*_, *α*_1_, *r*_1_, *r*_2_ > 0, and the prescribed parameters Δ_*q*_, *H*_*q*_, positive parameters *R*, arbitrary given parameters E and F with proper dimensions, positive constants $\bar{\varepsilon }$, *δ*, *c*_1_, *c*_2_, T, ensuring that the subsequent conditions are meet

$$\begin{array}{c} \begin{array}{cc} & [\begin{array}{cccc} {Ϝ}_{11} & {Ϝ}_{12} & {Ϝ}_{13} & {Ϝ}_{14}\\ {}* & {Ϝ}_{22} & {Ϝ}_{23} & {Ϝ}_{24}\\ {}* & * & {Ϝ}_{33} & {Ϝ}_{34}\\ {}* & * & * & {Ϝ}_{44} \end{array}]<0, \end{array} \end{array}$$ (19)

$$\begin{array}{c} \begin{array}{c} c_{2}<\frac{e^{-\delta T}\left({\bar{\lambda }}_{1}+{\bar{\lambda }}_{2}\right)c_{1}+\bar{\omega }\alpha }{{\underline{\lambda }}_{1}},\forall t\in \left[0,T\right]. \end{array} \end{array}$$ (20)

where

${Ϝ}_{11}=[\begin{array}{ccc} -\frac{\pi^{2}}{4l^{2}}\left[P_{1}A+*\right] & \frac{\pi^{2}}{4l^{2}}\left[P_{1}A+*\right] & 0\\ {}* & \Phi_{1} & P_{1}B_{i}\bar{F}\\ {}* & * & -\frac{r_{2}}{l}I \end{array}]$ ,

${Ϝ}_{12}=[\begin{array}{ccc} 0 & 0 & 0\\ P_{1}C_{i} & P_{2B}^{T} & 0\\ 0 & 0 & 0 \end{array}]$ , ${Ϝ}_{13}=[\begin{array}{ccc} 0 & 0 & 0\\ EV & 0 & \bar{\varepsilon }\left[P_{1}B_{i}\bar{F}-EU\right]\\ 0 & 0 & 0 \end{array}]$,

${Ϝ}_{14}=[\begin{array}{cc} 0 & 0\\ 0 & \frac{2\Delta_{q}\rho_{x1}}{H}\\ 0 & 0 \end{array}]$ , ${Ϝ}_{22}=[\begin{array}{ccc} -\frac{\alpha }{l}I & 0 & 0\\ {}* & \Pi_{1} & P_{2B}\\ {}* & * & -r_{1}I \end{array}]$,

${Ϝ}_{23}=[\begin{array}{ccc} 0 & 0 & 0\\ 0 & {\left[FV\right]}^{T} & 0\\ 0 & 0 & 0 \end{array}]$ , ${Ϝ}_{24}=[\begin{array}{cc} 0 & 0\\ V^{T} & 0\\ 0 & 0 \end{array}]$,

${Ϝ}_{33}=[\begin{array}{ccc} -\frac{\alpha_{1}}{l}I & 0 & V^{T}\\ {}* & -\frac{r_{2}}{l}I & 0\\ {}* & * & -\bar{\varepsilon }\left[U_{1}+*\right] \end{array}]$ , ${Ϝ}_{34}=[\begin{array}{cc} 0 & 0\\ \bar{\varepsilon }\left[\frac{2\Delta_{q}\rho_{u2}}{H}I-FU\right] & 0\\ 0 & 0 \end{array}]$,

${Ϝ}_{44}=-diag\left\{\bar{\varepsilon }\left[U+*\right],r_{1}I\right\}$ ,

$\Phi_{1}=-\frac{\pi^{2}}{4l^{2}}\left[P_{1}A+*\right]+\left[P_{1}A_{i}+*\right]+\delta P_{1}$ ,

Π_1_ = [*P*_2_*A*_*d*_ + *] + *α*_1_*lI* + *δP*2,

${\underline{\lambda }}_{1}=\lambda_{min}\left(R^{-\frac{1}{2}}P_{1}R^{-\frac{1}{2}}\right)$ , ${\bar{\lambda }}_{1}=\lambda_{max}\left(R^{-\frac{1}{2}}P_{1}R^{-\frac{1}{2}}\right)$, ${\underline{\lambda }}_{2}=\lambda_{min}\left(R^{-\frac{1}{2}}P_{2}R^{-\frac{1}{2}}\right)$, ${\bar{\lambda }}_{2}=\lambda_{max}\left(R^{-\frac{1}{2}}P_{2}R^{-\frac{1}{2}}\right)$, and the dynamic parameter *μ*(*t*) is adjusted in real-time as follows:

$$\begin{array}{c} \begin{array}{cc} & \frac{\rho_{x}}{H_{q}}\left\|z\left(s,t\right)\right\|\leq \bar{\mu }\left(s,t\right)\leq \frac{2\rho_{x}}{H_{q}}\left\|z\left(s,t\right)\right\|,\\ & \frac{\rho_{u}}{H_{q}}\|\int_{0}^{l}C_{d}\phi \left(s,t\right)ds\|\leq \hat{\mu }\left(t\right)\leq \frac{2\rho_{u}}{H_{q}}\left\|\int_{0}^{l}C_{d}\phi \left(s,t\right)ds\right\|. \end{array} \end{array}$$ (21)

where $\rho_{x}=\frac{\rho_{x1}}{r_{1}}\geq 1$, $\rho_{u}=\frac{\rho_{u2}}{r_{2}}\geq 1$. Consequently, finite-time boundedness for system (4) is established under the control scheme (15) within a finite-time interval. And the dynamic control gain is $A_{d}=P_{2}^{-1}P_{2A}$, $B_{d}=P_{2}^{-1}P_{2B}$, $C_{d}=U_{i}^{-1}V_{i}$.

*Proof*. For the system represented by (4), according to the stability analize method used in [37, 38], choose the Lyapunov candidate function as follows:

$$\begin{array}{c} V\left(t\right)=V_{1}\left(t\right)+V_{2}\left(t\right), \end{array}$$ (22)

where $V_{1}\left(t\right)=\int_{0}^{l}z^{T}\left(s,t\right)P_{1}z\left(s,t\right)ds$, $V_{2}\left(t\right)=\int_{0}^{l}\phi^{T}\left(s,t\right)P_{2}\phi \left(s,t\right)ds$, *P*_1_ > 0 and *P*_2_ > 0 are undetermined matrices. Then the Lyapunov candidate function can be guaranteed to be positive, which is necessary for the stability analysis and control design of the studied system. By differentiating *V*_1_(*t*) with respect to time,

$$\begin{array}{c} \begin{array}{cc} E\{LV_{1}\left(t\right)\} & =\int_{0}^{l}z^{T}\left(s,t\right)P_{1}z_{t}\left(s,t\right)ds+\int_{0}^{l}z_{t}^{T}\left(s,t\right)P_{1}z\left(s,t\right)ds\\ & =[\int_{0}^{l}z^{T}\left(s,t\right)P_{1}Az_{ss}\left(s,t\right))ds+*]\\ & \;+\int_{0}^{l}z^{T}\left(s,t\right)[\sum_{i=1}^{\kappa }h_{i}P_{1}A_{i}+*]z\left(s,t\right)\\ & \;+[\int_{0}^{l}z^{T}\left(s,t\right)\sum_{i=1}^{\kappa }h_{i}P_{1}B_{i}E\left\{u\left(t\right)\right\}ds+*]\\ & \;+[\int_{0}^{l}z^{T}\left(s,t\right)\sum_{i=1}^{\kappa }h_{i}P_{1}C_{i}\omega \left(t\right)ds+*]. \end{array} \end{array}$$ (23)

Applying Lemma 4, take into account the conditions (2) and perform integration by parts,

$$\begin{array}{c} \begin{array}{cc} & \left[\int_{0}^{l}z^{T}\left(s,t\right)P_{1}Az_{ss}\left(s,t\right))ds+*\right]\\ & =[z^{T}\left(s,t\right)P_{1}Az_{ss}{\left(s,t\right)|}_{s=0}^{s=l}+*]\\ & \;-\int_{0}^{l}z_{s}^{T}\left(s,t\right)[P_{1}A+*]z_{s}\left(s,t\right)ds\\ & =-\int_{0}^{l}z_{s}^{T}\left(s,t\right)[P_{1}A_{i}+*]z_{s}\left(s,t\right)ds\\ & \leq -\int_{0}^{l}z^{T}\left(s,t\right)\left[\frac{\pi^{2}}{4l^{2}}P_{1}A+*\right]z\left(s,t\right)ds\\ & \;-\int_{0}^{l}z^{T}\left(0,t\right)\left[\frac{\pi^{2}}{4l^{2}}P_{1}A+*\right]z\left(0,t\right)ds\\ & \;+[\int_{0}^{l}z^{T}\left(s,t\right)\left[\frac{\pi^{2}}{4l^{2}}P_{1}A+*\right]z\left(0,t\right)ds+*]. \end{array} \end{array}$$ (24)

By Lemma 3,

$$\begin{array}{c} \begin{array}{cc} & \left[\int_{0}^{l}z^{T}\left(s,t\right)\sum_{i=1}^{\kappa }h_{i}P_{1}B_{i}E\left\{u\left(t\right)\right\}ds+*\right]\\ & =[\int_{0}^{l}z^{T}\left(s,t\right)\sum_{i=1}^{\kappa }h_{i}P_{1}B_{i}\bar{F}\int_{0}^{l}C_{d}\phi \left(s,t\right)dsds+*]\\ & \;+[\int_{0}^{l}z^{T}\left(s,t\right)\sum_{i=1}^{\kappa }h_{i}P_{1}B_{i}\bar{F}\hat{\vartheta }\left(t\right)ds+*]\\ & \leq \frac{\alpha_{1}}{l}\int_{0}^{l}z^{T}\left(s,t\right){\left[\sum_{i=1}^{\kappa }h_{i}P_{1}B_{i}\bar{F}C_{d}\right]}^{T}\left[\sum_{i=1}^{\kappa }h_{i}P_{1}B_{i}\bar{F}C_{d}\right]z\left(s,t\right)ds\\ & \;+\alpha_{1}l\int_{0}^{l}\phi^{T}\left(s,t\right)\phi \left(s,t\right)ds\\ & \;+[\int_{0}^{l}z^{T}\left(s,t\right)\sum_{i=1}^{\kappa }h_{i}P_{1}B_{i}\bar{F}\hat{\vartheta }\left(t\right)ds+*]. \end{array} \end{array}$$ (25)

By calculating the time derivative of *V*_2_(*t*),

$$\begin{array}{c} \begin{array}{cc} E\{LV_{2}\left(t\right)\} & =\int_{0}^{l}\phi^{T}\left(s,t\right)\left[P_{2}A_{d}+*\right]\phi_{t}\left(s,t\right)ds\\ & +[\int_{0}^{l}\phi^{T}\left(s,t\right)P_{2}B_{d}x\left(s,t\right))ds+*]\\ & +[\int_{0}^{l}\phi^{T}\left(s,t\right)P_{2}B_{d}\bar{\vartheta }\left(s,t\right))ds+*]. \end{array} \end{array}$$ (26)

Utilizing the adjusting rule given by (21) and (13), we obtain:

$$\begin{array}{c} \begin{array}{cc} & \|\frac{z(s,t)}{\bar{\mu }\left(s,t\right)}\|\leq H\Rightarrow \|q(\frac{z(s,t)}{\mu (s,t)})-\frac{z(s,t)}{\mu (s,t)}\|\;<\Delta_{q},\\ & \|\frac{\int_{0}^{l}C_{d}\phi \left(s,t\right)ds}{\hat{\mu }\left(s,t\right)}\|\leq H\Rightarrow \|q(\frac{\int_{0}^{l}C_{d}\phi \left(s,t\right)ds}{\hat{\mu }\left(s,t\right)})-\frac{\int_{0}^{l}C_{d}\phi \left(s,t\right)ds}{\hat{\mu }\left(s,t\right)}\|\;<\Delta_{q} \end{array} \end{array}$$ (27)

With Lemma 1, consider the homogeneity property of Euclidean norm to $\bar{\vartheta }\left(s,t\right)$ and $\hat{\vartheta }\left(t\right)$, for any positive scalars *r*_1_ and *r*_2_, by Lemma 1, the relationship can be derived:

$$\begin{array}{c} \begin{array}{cc} & \int_{0}^{l}r_{1}{\bar{\vartheta }}^{T}\left(s,t\right)\bar{\vartheta }\left(s,t\right)ds\leq \int_{0}^{l}\frac{4\Delta_{q}^{2}\rho_{x}^{2}r_{1}}{H^{2}}z^{T}\left(s,t\right)z\left(s,t\right)ds,\\ & r_{2}{\hat{\vartheta }}^{T}\left(t\right)\hat{\vartheta }\left(t\right)\leq \int_{0}^{l}\frac{4\Delta_{q}^{2}\rho_{u}^{2}r_{2}l}{H^{2}}\phi^{T}\left(s,t\right)C_{d}^{T}C_{d}\phi \left(s,t\right)ds. \end{array} \end{array}$$ (28)

By both adding and subtracting,

$$\int_{0}^{l}r_{1}{\bar{\vartheta }}^{T}\left(s,t\right)\bar{\vartheta }\left(s,t\right)ds+r_{2}{\hat{\vartheta }}^{T}\left(s,t\right)\hat{\vartheta }\left(s,t\right)$$

to E{LV(t)} arrives:

$$\begin{array}{c} \begin{array}{cc} & {E\{LV\left(t\right)+\delta V\left(t\right)-\alpha \|w\left(t\right)\|}^{2}\}\leq \int_{0}^{l}\sum_{i=1}^{\kappa }h_{i}{\hat{z}}^{T}\left(s,t\right){Ϝ}_{1}\hat{z}\left(s,t\right)ds, \end{array} \end{array}$$ (29)

where ${\bar{Ϝ}}_{1}=[\begin{array}{cc} {\bar{Ϝ}}_{11} & {\bar{Ϝ}}_{12}\\ {}* & {\bar{Ϝ}}_{22} \end{array}]$,

${\bar{Ϝ}}_{11}=[\begin{array}{ccc} -\frac{\pi^{2}}{4l^{2}}\left[P_{1}A+*\right] & \frac{\pi^{2}}{4l^{2}}\left[P_{1}A+*\right] & 0\\ {}* & {\bar{\Phi }}_{1} & P_{1}B_{i}\bar{F}\\ {}* & * & -\frac{r_{2}}{l}I \end{array}]$ ,

${\bar{Ϝ}}_{12}=[\begin{array}{ccc} 0 & 0 & 0\\ P_{1}C_{i} & {\left[P_{2}B_{d}\right]}^{T} & 0\\ 0 & 0 & 0 \end{array}]$ , ${\bar{Ϝ}}_{22}=[\begin{array}{ccc} -\frac{\alpha }{l}I & 0 & 0\\ {}* & {\bar{\Pi }}_{1} & P_{2}B_{d}\\ {}* & * & -r_{1}I \end{array}]$,

${\bar{\Phi }}_{1}=\left[P_{1}A_{i}+*\right]-\frac{\pi^{2}}{4l^{2}}\left[P_{1}A+*\right]+\delta P_{1}+\frac{\alpha_{1}}{l}{\left[\sum_{i=1}^{\kappa }h_{i}P_{1}B_{i}\bar{F}C_{d}\right]}^{T}\left[\sum_{i=1}^{\kappa }h_{i}P_{1}B_{i}\bar{F}C_{d}\right]+\frac{4\Delta_{q}^{2}\rho_{x}^{2}r_{1}}{H^{2}}I$ ,

$\Pi_{1}=\left[P_{2}A_{d}+*\right]+\delta P_{2}+l\alpha_{1}I+\frac{4\Delta_{q}^{2}\rho_{u}^{2}r_{2}}{H^{2}}C_{d}^{T}C_{d}$ ,

$\hat{z}\left(s,t\right)=col\left\{z\left(0,t\right),z\left(s,t\right),\hat{\vartheta }\left(s,t\right),\omega \left(t\right),\phi \left(s,t\right),\bar{\vartheta }\left(s,t\right)\right\}$ .

Let *C*_*d*_ = *U*^−1^*V*, which means $P_{1}B_{i}\bar{F}C_{d}=\left[P_{1}B_{i}\bar{F}-EU\right]U^{-1}V+EV$, $\frac{2\Delta_{q}\rho_{u}^{2}r_{2}}{H}C_{d}=\left[\frac{2\Delta_{q}\rho_{u}^{2}r_{2}}{H}I-FU\right]U^{-1}V+FV$, where E and F are any given matrices with proper dimension. Then under the Schur Complement, the matrix *Ϝ*_1_ can be expressed as:

$$\begin{array}{c} \begin{array}{c} {\tilde{Ϝ}}_{1}={\hat{Ϝ}}_{1}+\left[\Lambda_{11}diag\left\{U^{-1}V,U^{-1}V\right\}\Lambda_{12}+*\right], \end{array} \end{array}$$ (30)

where ${\hat{Ϝ}}_{1}=[\begin{array}{ccc} {\hat{Ϝ}}_{11} & {\bar{Ϝ}}_{12} & {\bar{Ϝ}}_{13}\\ {}* & {\hat{Ϝ}}_{22} & {\bar{Ϝ}}_{23}\\ {}* & * & {\bar{Ϝ}}_{33} \end{array}]$,

${\bar{Ϝ}}_{11}=[\begin{array}{ccc} -\frac{\pi^{2}}{4l^{2}}\left[P_{1}A+*\right] & \frac{\pi^{2}}{4l^{2}}\left[P_{1}A+*\right] & 0\\ {}* & {\hat{\Phi }}_{1} & P_{1}B_{i}\bar{F}\\ {}* & * & -\frac{r_{2}}{l}I \end{array}]$ ,

${\bar{Ϝ}}_{13}=[\begin{array}{cc} 0 & 0\\ EV & 0\\ 0 & 0 \end{array}]$ , ${\bar{Ϝ}}_{22}=[\begin{array}{ccc} -\frac{\alpha }{l}I & 0 & 0\\ {}* & {\hat{\Pi }}_{1} & P_{2}B_{d}\\ {}* & * & -r_{1}I \end{array}]$, ${\bar{Ϝ}}_{23}=[\begin{array}{cc} 0 & 0\\ 0 & {\left[FV\right]}^{T}\\ 0 & 0 \end{array}]$,

${\hat{\Phi }}_{1}=\left[P_{1}A_{i}+*\right]-\frac{\pi^{2}}{4l^{2}}\left[P_{1}A+*\right]+\delta P_{1}+\frac{4\Delta_{q}^{2}\rho_{x}^{2}r_{1}}{H^{2}}I$ ,

Π_1_ = [*P*_2_*A*_*d*_ + *] + *δP*_2_ + *lα*_1_*I*.

$\Lambda_{11}^{T}=[\begin{array}{cccccccc} 0 & {\left[P_{1}B_{i}\bar{F}-EU\right]}^{T} & 0 & 0 & 0 & 0 & 0 & 0\\ 0 & 0 & 0 & 0 & 0 & 0 & 0 & {\left[\frac{2\Delta_{q}\rho_{x}r_{2}}{H}I-FU\right]}^{T} \end{array}{]}^{T}$ ,

$\Lambda_{12}=[\begin{array}{cccccccc} 0 & 0 & 0 & 0 & 0 & 0 & I & 0\\ 0 & 0 & 0 & 0 & 0 & I & 0 & 0 \end{array}]$ .

If *Ϝ*_1_ < 0, let *P*_2_*B*_*d*_ = *P*_2*B*_, condition (20) can be deduced as

$$\begin{array}{c} \begin{array}{c} E\{LV\left(t\right)+\delta V\left(t\right)-\alpha \|\omega \left(t\right){\|}^{2}\}<0. \end{array} \end{array}$$ (31)

by using the Lemma 2 as well as the basic Schur complement method Integrating (31) from 0 to T while considering the initial value condition, we can obtain:

$$\begin{array}{c} \begin{array}{c} e^{\delta T}EV\left(T\right)-V\left(0\right)<\int_{0}^{T}e^{\delta t}\alpha {\left\|\omega \left(t\right)\right\|}^{2}dt. \end{array} \end{array}$$ (32)

Inequality (32) further means:

$$\begin{array}{c} \begin{array}{cc} E\{V(T)\} & <e^{-\delta T}[V\left(0\right)+\int_{0}^{T}e^{\delta t}\alpha {\left\|\omega \left(t\right)\right\|}^{2}dt]\\ & <e^{-\delta T}[\int_{\Omega }z^{T}\left(s,0\right)P_{1}z\left(s,0\right)ds+\int_{\Omega }\phi^{T}\left(s,0\right)P_{2}\phi \left(s,0\right)ds+e^{-\delta T}\bar{\omega }\alpha ]\\ & =e^{-\delta T}[\int_{\Omega }z^{T}\left(s,0\right)R^{\frac{1}{2}}\left(R^{-\frac{1}{2}}P_{1}R^{-\frac{1}{2}}\right)R^{\frac{1}{2}}z\left(s,0\right)ds\\ & \;+\int_{\Omega }\phi^{T}\left(s,0\right)R^{\frac{1}{2}}\left(R^{-\frac{1}{2}}P_{2}R^{-\frac{1}{2}}\right)R^{\frac{1}{2}}\phi \left(s,0\right)ds+e^{-\delta T}\bar{\omega }\alpha ]\\ & <e^{-\delta T}\left[{\bar{\lambda }}_{1}\int_{\Omega }z^{T}\left(s,0\right)Rz\left(s,0\right)ds+{\bar{\lambda }}_{2}\int_{\Omega }\phi^{T}\left(s,0\right)R\phi \left(s,0\right)ds\right]+\bar{\omega }\alpha \\ & <e^{-\delta T}\left({\bar{\lambda }}_{1}+{\bar{\lambda }}_{2}\right)c_{1}+\bar{\omega }\alpha . \end{array} \end{array}$$ (33)

Conversely,

$$\begin{array}{c} \begin{array}{cc} E\{V(T)\} & =\int_{\Omega }z^{T}\left(s,T\right)Pz\left(s,T\right)ds\\ & =\int_{\Omega }z^{T}\left(s,T\right)R^{\frac{1}{2}}\left(R^{-\frac{1}{2}}P_{1}R^{-\frac{1}{2}}\right)R^{\frac{1}{2}}z\left(s,T\right)ds\\ & >{\underline{\lambda }}_{1}\int_{\Omega }z^{T}\left(s,T\right)Rz\left(s,T\right)ds. \end{array} \end{array}$$ (34)

Then the following relationship is derived,

$$\begin{array}{c} \begin{array}{c} \int_{\Omega }z^{T}\left(s,t\right)Rz\left(s,t\right)ds<\frac{e^{-\delta T}\left({\bar{\lambda }}_{1}+{\bar{\lambda }}_{2}\right)c_{1}+\bar{\omega }\alpha }{{\underline{\lambda }}_{1}},\forall t\in \left[0,T\right]. \end{array} \end{array}$$ (35)

Utilizing (20), it can be established that ∫_*Ω*_ *z*^*T*^(*s*, *t*)*Rz*(*s*, *t*)*ds* < *c*_2_.

As a result, the Finite-Time Boundedness (FTB) of system (4) is assured through the use of controller (15). This concludes the proof.

**Theorem 2**. Considering the closed-loop system represented by Eq (4), for the undetermined positive parameters *P*_1_ and *P*_2_, properly parameters *P*_2*A*_, *P*_2*B*_, *V*, positive scalars *r*_1_, *r*_2_, and *α*_1_, if the conditions (19)-(20) and the following inequality are satisfied with the quadratic energy supply function (6) and the prescribed parameters *ρ*_*u*_, *ρ*_*y*_, $\bar{\varepsilon }$, *δ*, *c*_1_, *c*_2_, T:

$$\begin{array}{c} \begin{array}{cc} {Ϝ}_{2}= & [\begin{array}{cccc} {\tilde{Ϝ}}_{11} & {\tilde{Ϝ}}_{12} & {Ϝ}_{13} & {Ϝ}_{14}\\ {}* & {\tilde{Ϝ}}_{22} & {Ϝ}_{23} & {Ϝ}_{24}\\ {}* & * & {Ϝ}_{33} & {Ϝ}_{34}\\ {}* & * & * & {Ϝ}_{44} \end{array}]<0, \end{array} \end{array}$$ (36)

${\tilde{Ϝ}}_{11}=[\begin{array}{ccc} -\frac{\pi^{2}}{4l^{2}}\left[P_{1}A+*\right]-D^{T}J_{1}D & \frac{\pi^{2}}{4l^{2}}\left[P_{1}A+*\right] & 0\\ {}* & \Phi_{1} & P_{1}B_{i}\bar{F}\\ {}* & * & -\frac{r_{2}}{l}I \end{array}]$ ,

${\tilde{Ϝ}}_{12}=[\begin{array}{ccc} D^{T}J_{2} & 0 & 0\\ P_{1}C_{i} & P_{2B}^{T} & 0\\ 0 & 0 & 0 \end{array}]$ , ${\tilde{Ϝ}}_{22}=[\begin{array}{ccc} \frac{\alpha }{l}I-\frac{J_{3}}{l} & 0 & 0\\ {}* & \Pi_{1} & P_{2B}\\ {}* & * & -r_{1}I \end{array}]$, and the dynamic parameters $\bar{\mu }\left(s,t\right)$ and $\hat{\mu }\left(t\right)$ are updated in real-time according to (21), then it can be affirmed that the system (4), governed by *J*(*t*), exhibits EDFTB with regard to the parameters (*c*_1_, *c*_2_, T, *α*, *J*_1_, *J*_2_, *J*_3_).

*Proof*: Using the function *V*(*t*) for the fuzzy DP-CPS described in (4), and considering the quadratic energy supply function given in (6), we can establish the following inequality through Theorem 1:

$$\begin{array}{c} \begin{array}{cc} & E\left\{LV\left(t\right)-\delta V\left(t\right)-J\left(t\right)+\alpha \omega^{T}\left(t\right)\omega \left(t\right)\right\}\leq \int_{0}^{l}{\hat{z}}^{T}\left(s,t\right){Ϝ}_{2}\hat{z}\left(s,t\right)ds. \end{array} \end{array}$$ (37)

If inequality (36) as stated in Theorem 2 holds, then:

$$\begin{array}{c} \begin{array}{c} E\{LV\left(t\right)-\delta V\left(t\right)-J\left(t\right)+\alpha \|\omega \left(t\right){\|}^{2}\}\leq 0. \end{array} \end{array}$$ (38)

If the inequality (36) presented in Theorem 2 is valid, then integrate from 0 to T arrives:

$$\begin{array}{c} \begin{array}{c} e^{-\delta T}E\left\{V\left(T\right)\right\}-E\left\{V\left(0\right)\right\}<\int_{0}^{T}\left[J\left(t\right)-\alpha \omega^{T}\left(t\right)\omega \left(t\right)\right]dt. \end{array} \end{array}$$ (39)

Consider $EV(0)=-\hat{\alpha }$, since $e^{-\delta T}E\left\{V\left(T\right)\right\}>0$, then

$$\begin{array}{c} \begin{array}{c} \int_{0}^{T}J\left(t\right)dt\geq \alpha \int_{0}^{T}\omega^{T}\left(t\right)\omega \left(t\right)dt+\hat{\alpha }. \end{array} \end{array}$$ (40)

Through the utilization of Definition 2 and the results from Theorem 1, we have successfully addressed the controller design problem for achieving EDFTB of DP-CPS (4). This concludes the proof.

**Remark 2**. The dynamic parameter $\bar{\mu }$ and $\hat{\mu }$, which are transmitted through the communication channel, is adjusted according to the rule outlined in [16].

***The adjusting rule***:

$$\begin{array}{c} \begin{array}{c} \bar{\mu }=\{\begin{array}{cc} & floor(\frac{2\rho_{x}}{H}\left|z\left(s,t\right)\right|\times 10^{\ell })\times 10^{-\ell },\;\;0\leq \frac{2\rho_{x}}{H}\left|z\left(s,t\right)\right|<\frac{1}{2},\\ & 1,\;\;\frac{1}{2}\leq \frac{\rho_{x}}{H}\left|z\left(s,t\right)\right|<1,\\ & floor(\frac{\rho_{x}}{H}\left|z\left(s,t\right)\right|),\;\;1<\frac{\rho_{x}}{H}\left|z\left(s,t\right)\right|, \end{array} \end{array} \end{array}$$ (41)

$$\begin{array}{c} \begin{array}{c} \hat{\mu }=\{\begin{array}{cc} & floor(\frac{2\rho_{u}}{H}\left|\int_{0}^{l}C_{d}\phi \left(s,t\right)ds\right|\times 10^{\ell })\times 10^{-\ell },\;\;0\leq \frac{2\rho_{u}}{H}\left|\int_{0}^{l}C_{d}\phi \left(s,t\right)ds\right|<\frac{1}{2},\\ & 1,\;\;\frac{1}{2}\leq \frac{\rho_{u}}{H}\left|\int_{0}^{l}C_{d}\phi \left(s,t\right)ds\right|<1,\\ & floor(\frac{\rho_{u}}{H}\left|\int_{0}^{l}C_{d}\phi \left(s,t\right)ds\right|),\;\;1<\frac{\rho_{u}}{H}\left|\int_{0}^{l}C_{d}\phi \left(s,t\right)ds\right|, \end{array} \end{array} \end{array}$$ (42)

here, the *floor*(*ζ*) means the largest integer of *ζ*, but less than *ζ*, and $\ell =min\{\ell \in N^{+}|\frac{2\rho_{u}}{H}|x\left(s,t\right)|\times 10^{\ell }>1\}$.

***Algorithm for application***:

*step 1*: Choose the parameters Δ_*q*_, *H*_*q*_, *ε*, E, F, *α*, *R*, *c*_1_, *c*_2_, T, and *δ*;

*step 2*: By the system parameters A(z(s,t)), *B*(*s*), *C*(*s*), *D*, and *l*, solve the LMIs in Thoerem 1 and Theorem 2 to find sufficient solutions of the controller parameters *A*_*d*_, *B*_*d*_, *C*_*d*_, *ρ*_*x*_, and *ρ*_*u*_; otherwise, return to Step 1;

*step 3*: Take the parameters into the controller form (15) derives the control input signal;

*step 4*: Transmit the control input signal to the system (1) derives the system control results, stop.

## Simulation studies

**Fig 2:**
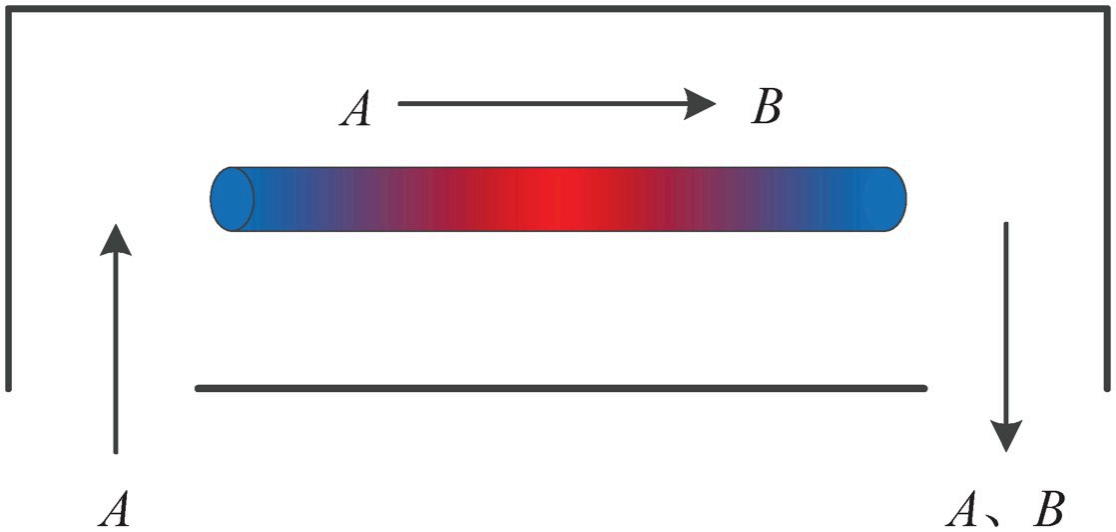
Catalytic rod in a reactor.

Consider a furnace which is filled with specie *A* and a catalytic reaction of the form *A* → *B* takes place on a long thin rod shown in Fig 2. This rod acts as a cooling medium since the reaction in the furnace is exothermic. Consider the case where the cooling process is two cylindrical catalytic rods coupled to each other. Similar to [39], take into account system (1) with the following set of parameters for the convenience of simulation:

$$\begin{array}{c} \begin{array}{cc} & A=[\begin{array}{cc} 1 & 0\\ 0 & 1 \end{array}],B\left(s\right)=B=[\begin{array}{cc} -0.35 & 0\\ 0 & -0.77 \end{array}],\\ & C\left(s\right)=C=[\begin{array}{cc} 0.96 & 1.2\\ 0.8 & 0.48 \end{array}],D=[\begin{array}{cc} 0.08 & 0.15\\ -0.2 & -0.13 \end{array}],l=1. \end{array} \end{array}$$ (43)

The state *z*(*s*, *t*) is the dimensionless temperature of the catalytic rod. *f*(*z*(*s*, *t*), *s*) is the nonlinear function related to the the heat, the activation energy, and the transfer coefficient of heat in the reaction.*y*(*t*) denotes the measured output, *u*(*t*) and *ω*(*t*) denote the control input and the related disturbance.*B*(*s*) is the distribution matrix of control actuators, *C*(*s*) is a known constant matrix, *l* is the length of the catalytic rod.

In this system, we consider some boundary conditions and initial states as follows:

$$\begin{array}{c} \begin{array}{cc} & z_{s}\left(s,t\right){{|}_{s=0}=z_{s}\left(s,t\right)|}_{s=l}=0,\\ & z_{0}\left(s\right)=[\begin{array}{c} 0.2-0.2\;\text{cos}(2\pi s)\\ 0.1+0.4\;\text{cos}(2\pi s) \end{array}], \end{array} \end{array}$$ (44)

For simulation convenience, select the nonlinear function

$$f\left(z\left(s,t\right)\right)=[\begin{array}{c} 0.1445z_{1}\left(s,t\right)+1.0605z_{2}\left(s,t\right)\\ -1.3z_{1}\left(s,t\right)-z_{2}^{3}\left(s,t\right)+0.01z_{2}\left(s,t\right) \end{array}].$$

**Fig 3:**
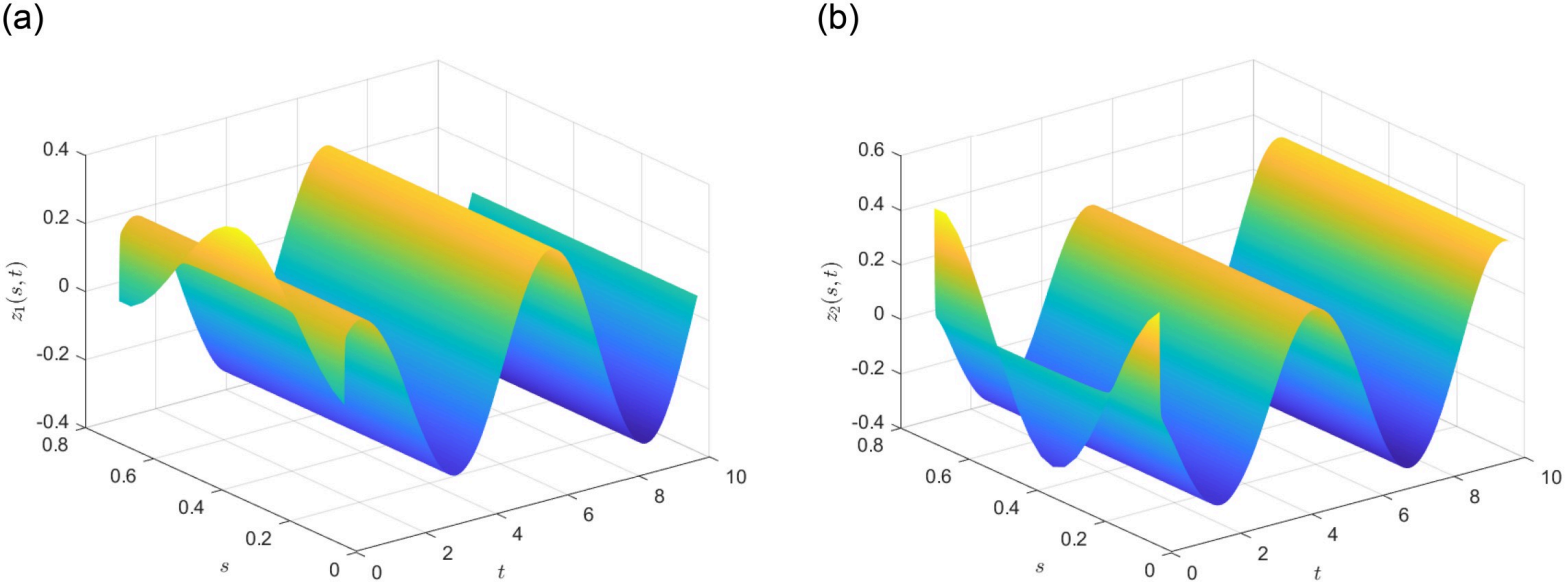
State profile of open loop DP-CPS.

Subsequently, the system state without control is determined and illustrated in Fig 3 in the presence of

$$\omega \left(t\right)=[\begin{array}{c} 0.02e^{-0.5t}+0.01e^{-0.5t}\;\text{cos}\left(2\pi t\right)+0.01e^{-0.5t}\;\text{sin}\left(3\pi t\right)\\ 0.01e^{-0.5t}+0.02e^{-0.5t}\;\text{sin}\left(2\pi t\right)+0.01e^{-0.5t}\;\text{cos}\left(3\pi t\right) \end{array}].$$

with $\bar{\omega }=1$. It is evident that the DP-CPS (1)-(2) exhibit instability. Based on the simulation results shown in Fig 3, the local domain of system state can be defined as $D=[I_{min},I_{max}^{1}]$ with the constraints $I_{min}\leq -1$ and $I_{min}\geq 1$. Choose $\varkappa \left(s,t\right)=z_{1}^{2}\left(s,t\right)$ and make the assumption that $\varkappa \left(s,t\right)=h_{1}\left(\varkappa \left(s,t\right)\right)I_{max}+h_{2}\left(\varkappa \left(s,t\right)\right)I_{min}$. Consequently, we obtain:

$$\begin{array}{c} \begin{array}{c} h_{1}\left(\varkappa \left(s,t\right)\right)=\frac{\varkappa \left(s,t\right)-I_{min}}{I_{max}-I_{min}},h_{2}\left(\varkappa \left(s,t\right)\right)=\frac{I_{max}-\varkappa \left(s,t\right)}{I_{max}-I_{min}}, \end{array} \end{array}$$ (45)

The function *f*(*z*(*s*, *t*)) is expressed as:

$$\begin{array}{c} \begin{array}{c} f\left(x\left(s,t\right)\right)=\sum_{i=1}^{2}h_{i}A_{i}z\left(s,t\right), \end{array} \end{array}$$ (46)

where $A_{1}=[\begin{array}{cc} 0.1445 & 1.0605\\ -0.3 & 0.01-I_{max} \end{array}]$, $A_{2}=[\begin{array}{cc} 0.1445 & 1.0605\\ -0.3 & 0.01-I_{min} \end{array}]$.

Define the fuzzy sets as “Small” and “Big”. The fuzzy rules are given as:

Plant rule 1: **IF** *ϰ*(*s*, *t*) is “Big”, **THEN**

$$z_{t}\left(s,t\right)=Az_{ss}\left(s,t\right)+A_{1}z\left(s,t\right)+Bu\left(t\right)+C\omega \left(t\right).$$ (47)

Plant rule 2: **IF** *ϰ*(*s*, *t*) is “Small”, **THEN**

$$z_{t}\left(s,t\right)=Az_{ss}\left(s,t\right)+A_{2}z\left(s,t\right)+Bu\left(t\right)+C\omega \left(t\right).$$ (48)

Then, the overall fuzzy model is derived:

$$z_{t}\left(s,t\right)=Az_{ss}\left(s,t\right)+\sum_{i=1}^{2}h_{i}\left(\varkappa \left(s,t\right)\right)\left[A_{i}z\left(s,t\right)+Bu\left(s,t\right)+C\omega \left(t\right)\right],$$ (49)

The signals $\int_{0}^{l}C_{d}\phi \left(s,t\right)ds$ and state *z*(*s*, *t*) are quantized by a class of dynamic quantizers to deal with the limited capability of communication channels. With the rule of quantizer (14) and the adjusting rule presented in Remark 1, the signals are quantized on the encoder side and the quantized values are decoded on the decoder side.

**Fig 4:**
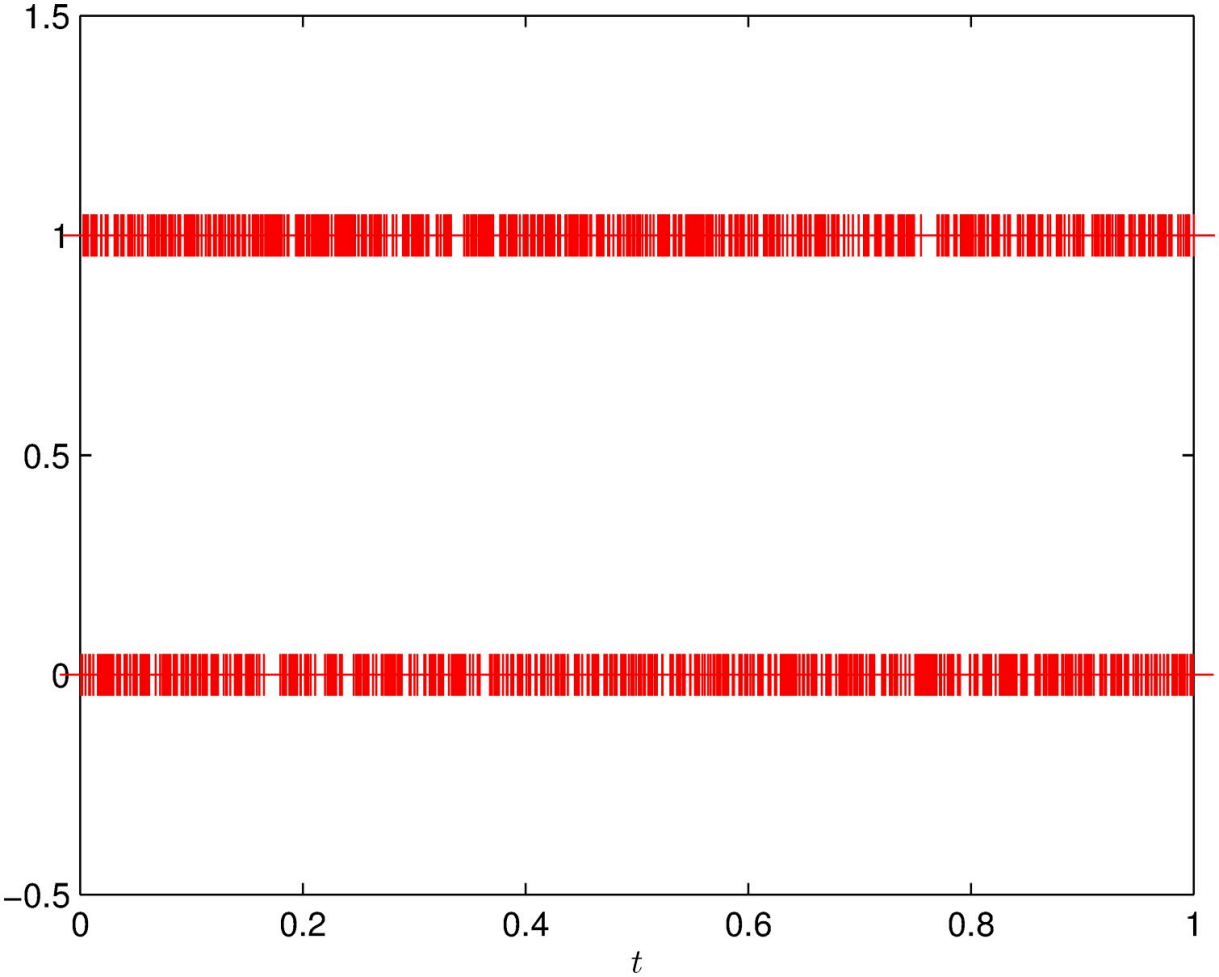
Stochastic process of Bernoulli with *F*(*t*).

In practical applications, the quantizer’s error Δ_*q*_ is chosen based on the limited bandwidth of the communication network, the quantization range ℵ is determined through the quantized information. By Fig 3, select the error and range of quantizaer as Δ_*q*_ = 0.001 as *H*_*q*_ = 100. Additionally, we have the following matrix and scalar values: *ε* = 0.1, E=I, F=2.5I, *α* = 0.75, *R* = *I*, *c*_1_ = 0.4, *c*_2_ = 150, T=10, *δ* = 0.0001. In Fig 4, the Bernoulli process with a mathematical expectation of $\bar{F}=0.5$ is presented. To assess the efficacy of the control strategy, the quadratic energy supply function parameters are as follows: $J_{1}=[\begin{array}{cc} 1 & 0\\ 0 & 2 \end{array}]$, $J_{2}=[\begin{array}{cc} 1 & 2\\ 3 & 2 \end{array}]$, and $J_{3}=[\begin{array}{cc} 2 & 0\\ 0 & 1 \end{array}]$, By solving the controller design conditions outlined in Theorem 2, we can derive the feasible parameters as follows:

$$\begin{array}{c} \begin{array}{cc} & A_{d}=[\begin{array}{cc} -9.4976 & 0.0983\\ 0.0983 & -10.0265 \end{array}],B_{d}=[\begin{array}{cc} 0.1667 & 0\\ 0 & 0.1667 \end{array}],\\ & C_{d}=[\begin{array}{cc} 1.4575 & 0.0006\\ 0.0005 & 1.5357 \end{array}],\rho_{x}=1.9999,\rho_{u}=2.4761,\\ & P_{1}=[\begin{array}{cc} 0.0881 & 0.0009\\ 0.0009 & 0.0835 \end{array}],P_{2}=[\begin{array}{cc} 1.1198 & 0\\ 0 & 1.1198 \end{array}],\\ & \alpha_{1}=0.7839,r_{1}=1.1198,r_{2}=0.7864. \end{array} \end{array}$$ (50)

**Fig 5:**
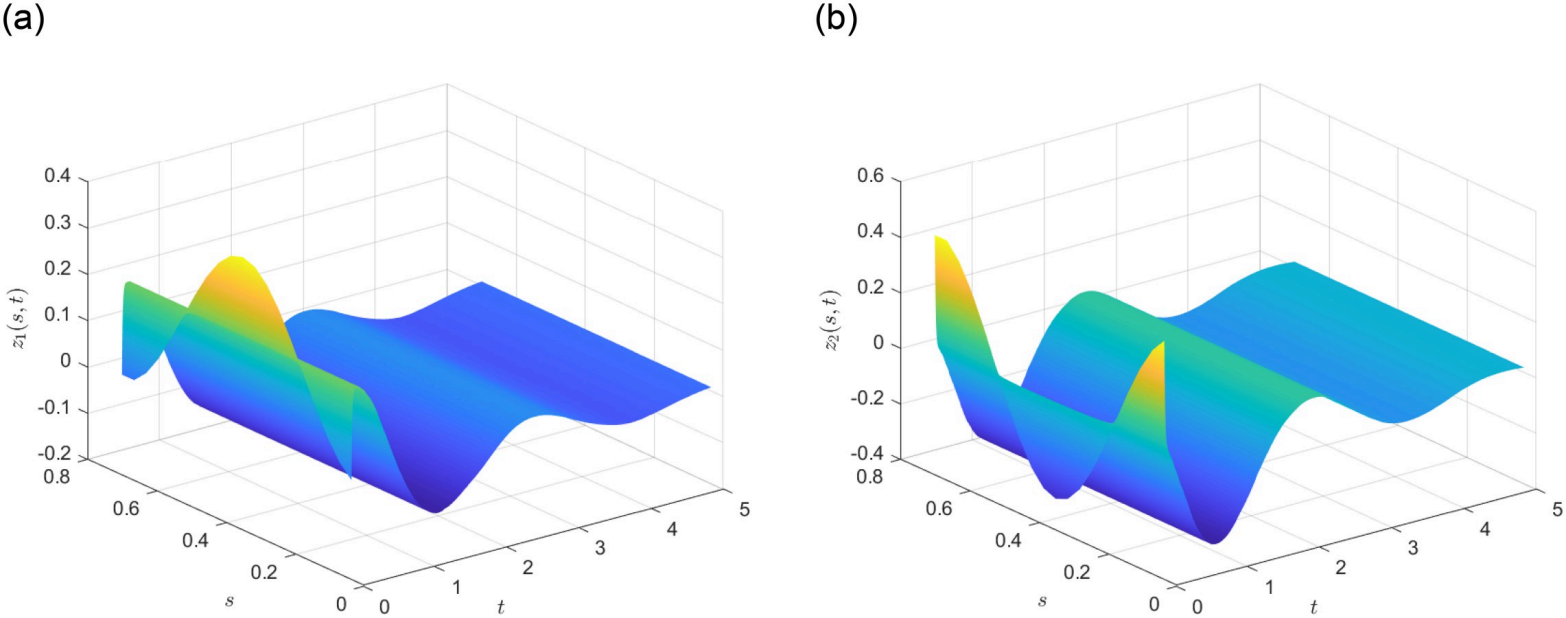
State profile of close loop DP-CPS.

**Fig 6:**
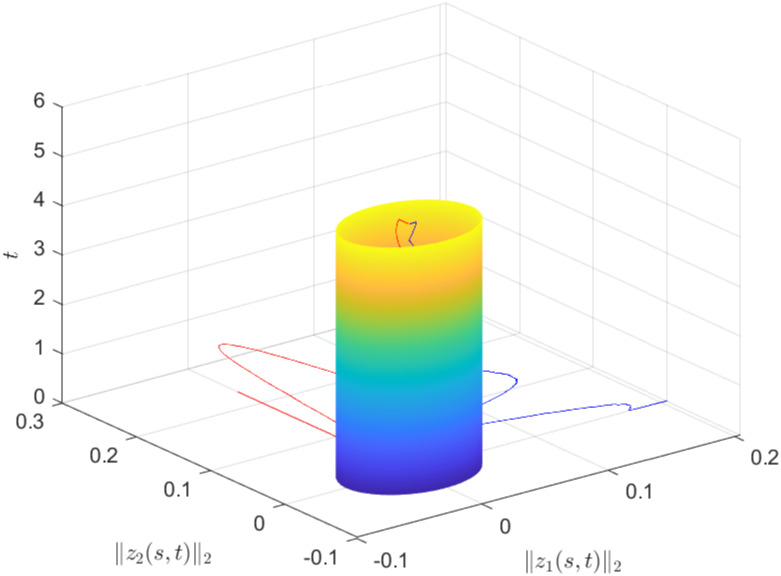
‖*z*_*i*_(*s*, *t*)‖_2_(*i* = 1, 2).

**Fig 7:**
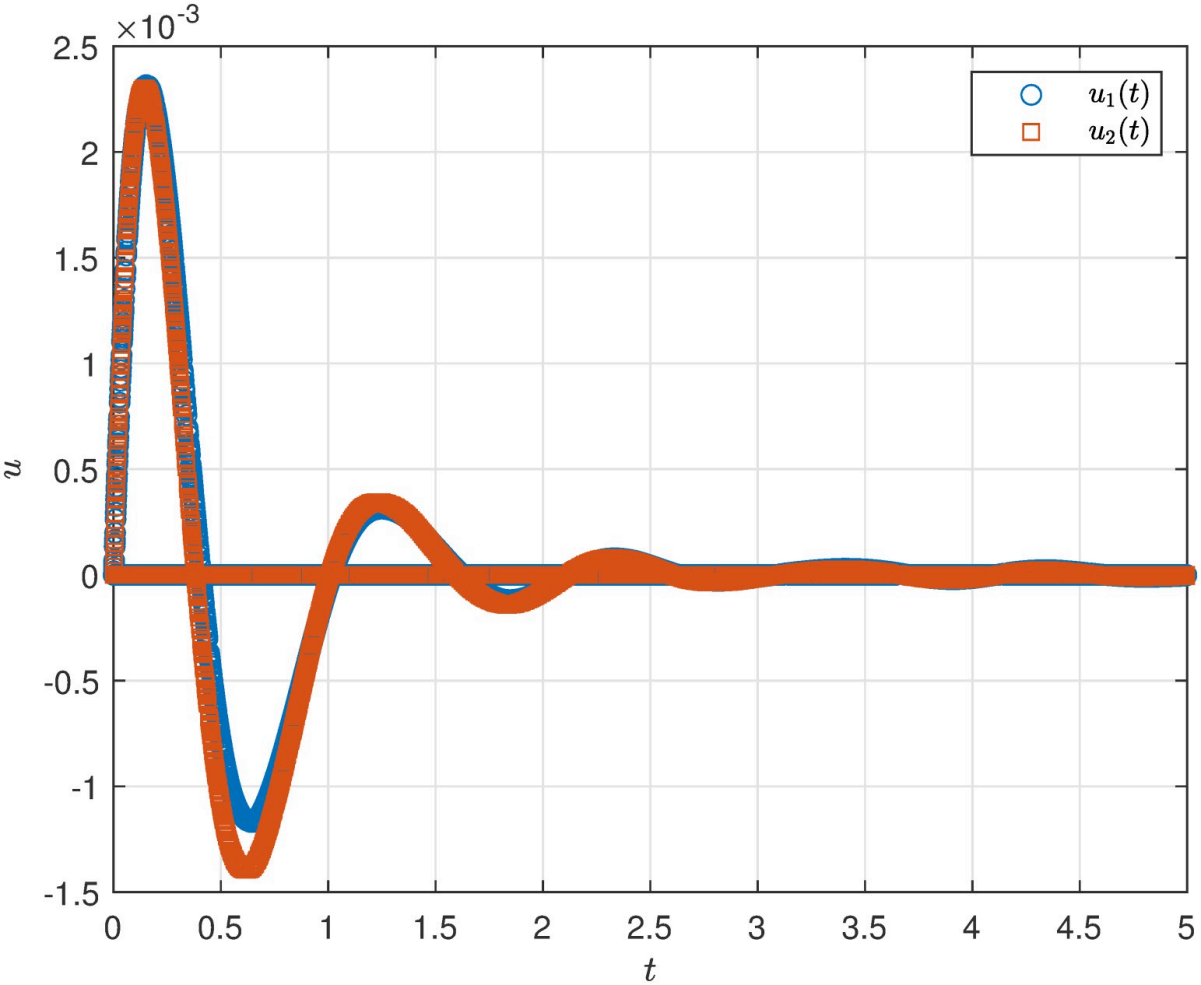
Controller simulation *u*(*t*).

The state profile of the considered close loop DP-CPS under (15) is demonstrated by Fig 5. The evolution of the norms ‖*x*_1_(*s*, *t*)‖_2_ and ‖*x*_2_(*s*, *t*)‖_2_ under (15) can be seen in Fig 6, while Fig 7 illustrates the simulations of *u*_1_(*t*) and *u*_2_(*t*).

The simulations clearly indicate that the designed control method under the influence of DoS attack, effectively achieves the specified performance for the DP-CPS.

**Fig 8:**
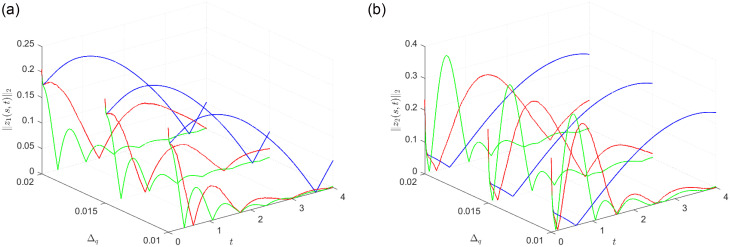
‖*z*_*i*_(*s*, *t*)‖_2_(*i* = 1, 2) with different Δ_*q*_.

Furthermore, to evaluate the robustness of the control design method, Fig 8 presents the simulations and the comparisons under varying levels of quantization error bounds Δ_*q*_. The figure legend is as follows: The blue line represents the simulation of the control strategy with a static quantizer, as presented in [12]. The green line illustrates the simulation without signal quantization. The red line showcases the simulation results of the control strategy introduced in this article. Compared with literature [12], the control method presented in this paper is more superior.

The simulation results confirm that the control strategies presented in this study effectively ensure the EDFTB of the analyzed system.

## Conclusion

In this study, a finite-time dissipative control strategy for nonlinear distributed parameter CPS modeled as parabolic PDEs is investigated by utilizing the T-S fuzzy model. Employing the dynamic quantizer, the system state and input signal have been quantized to alleviate transmission pressures and simplify quantization rules. Taking into account potential DoS attack stemming from the application of communication, a dynamic state feedback controller under attack has been designed. Subsequently, conditions ensuring finite-time boundedness and dissipative performance for the designed controller, as well as the parameter of the quantizer, have been established. The efficacy of the proposed dissipative control methodology has been validated through simulations. However, the control strategies proposed in this study haven’t considered the fact that the control input is usually constrained in a finite region. Future research will focus on the finite-time control design of the studied system with input constraint.

## Supporting information

S1 File The code. (DOCX)
