# Supplementary material for: Dynamic dissipative control for fuzzy distributed parameter cyber physical system under input quantization and DoS attack
Source: PLoS One. 2024 Oct 3;19(10):e0311215. doi: 10.1371/journal.pone.0311215 (PMC11449298; doi:10.1371/journal.pone.0311215)
Supplement: S1 File — (DOCX) [file pone.0311215.s001.docx]

**The code**

clear all;

clc;

t_max=10;

s_max=0.7;

L=s_max;

Det_q=0.02;

H=100;

bar_F=0.5;

det=0.005;

bar_emp=5.3;

math_E=0.5*[1,0;0,2];

math_F=2.5*[1,0;0,1];

math_A=1*[1,0;0,1];

a_11 =0.1445;

a_12 =1.0605;

a_21 =-0.3;

a_22 =0.01;

wep_max=1;

wep_min=-1;

A1=[a_11, a_12; a_21, a_22-wep_max];

A3=[a_11, a_12; a_21, a_22-wep_min];

A2=A1;

A4=A3;

B=[ -0.35,0;0,-0.77];

C=0.4*[ 2.4,3;2,1.2];

D=[0.08, 0.15; -0.2, -0.13];

J1=[1,0;0,2];

J2=[1,2;3,2];

J3=[2,0;0,1];

setlmis([])

P1=lmivar(1,[2 1]);

P2=lmivar(1,[2 1]);

P2A=lmivar(2,[2 2]);

P2B=lmivar(2,[2 2]);

U=lmivar(2,[2 2]);

V=lmivar(2,[2 2]);

rou_x1=lmivar(1,[1 1]);

rou_u2=lmivar(1,[1 1]);

r1=lmivar(1,[1 1]);

r2=lmivar(1,[1 1]);

alpha1=lmivar(1,[1 1]);

alpha=lmivar(1,[1 1]);

lmiterm([1 1 1 P1], -pi^2/(4*L^2), math_A, 's');

lmiterm([1 1 2 P1], pi^2/(4*L^2), math_A, 's');

lmiterm([1 1 4 0], D'*J2);

lmiterm([1 2 2 P1], -pi^2/(4*L^2), math_A, 's');

lmiterm([1 2 2 P1], 1, A1, 's');

lmiterm([1 2 2 P1], det, 1);

lmiterm([1 2 3 P1], 1, B*bar_F);

lmiterm([1 2 4 P1], 1, C);

lmiterm([1 2 5 -P2B], 1, 1);

lmiterm([1 2 7 V], math_E, 1);

lmiterm([1 2 9 P1], bar_emp, B*bar_F);

lmiterm([1 2 9 U], -bar_emp*math_E, 1);

lmiterm([1 2 11 rou_x1], 2*Det_q/H,1);

lmiterm([1 3 3 r2], -1/L, 1);

lmiterm([1 4 4 0], -J3);

lmiterm([1 4 4 alpha], 1,1);

lmiterm([1 5 5 P2A], 1, 1, 's');

lmiterm([1 5 5 P2], det, 1);

lmiterm([1 5 5 alpha1], L, 1);

lmiterm([1 5 6 P2B], 1, 1);

lmiterm([1 5 8 -V], 1, math_F');

lmiterm([1 5 10 -V], 1, 1);

lmiterm([1 6 6 r1], -1, 1);

lmiterm([1 7 7 alpha1], -1/L, 1);

lmiterm([1 7 9 -V], 1, 1);

lmiterm([1 8 8 r2], -1/L, 1);

lmiterm([1 8 10 rou_u2], bar_emp, 2*Det_q/H);

lmiterm([1 8 10 U], -bar_emp*math_F, 1);

lmiterm([1 9 9 U], -bar_emp, 1, 's');

lmiterm([1 10 10 U], -bar_emp, 1, 's');

lmiterm([1 11 11 r1], -1, 1);

lmiterm([2 1 1 P1], -pi^2/(4*L^2), math_A, 's');

lmiterm([2 1 2 P1], pi^2/(4*L^2), math_A, 's');

lmiterm([2 2 2 P1], -pi^2/(4*L^2), math_A, 's');

lmiterm([2 2 2 P1], 1, A3, 's');

lmiterm([2 2 2 P1], det, 1);

lmiterm([2 2 3 P1], 1, B*bar_F);

lmiterm([2 2 4 P1], 1, C);

lmiterm([2 2 5 -P2B], 1, 1);

lmiterm([2 2 7 V], math_E, 1);

lmiterm([2 2 9 P1], bar_emp, B*bar_F);

lmiterm([2 2 9 U], -bar_emp*math_E, 1);

lmiterm([2 2 11 rou_x1], 2*Det_q/H,1);

lmiterm([2 3 3 r2], -1/L, 1);

lmiterm([2 4 4 alpha], -1,1);

lmiterm([2 5 5 P2A], 1, 1, 's');

lmiterm([2 5 5 P2], det, 1);

lmiterm([2 5 5 alpha1], L, 1);

lmiterm([2 5 6 P2B], 1, 1);

lmiterm([2 5 8 -V], 1, math_F');

lmiterm([2 5 10 -V], 1, 1);

lmiterm([2 6 6 r1], -1, 1);

lmiterm([2 7 7 alpha1], -1/L, 1);

lmiterm([2 7 9 -V], 1, 1);

lmiterm([2 8 8 r2], -1/L, 1);

lmiterm([2 8 10 rou_u2], bar_emp, 2*Det_q/H);

lmiterm([2 8 10 U], -bar_emp*math_F, 1);

lmiterm([2 9 9 U], -bar_emp, 1, 's');

lmiterm([2 10 10 U], -bar_emp, 1, 's');

lmiterm([2 11 11 r1], -1, 1);

lmiterm([3 1 1 P1], -1, 1);

lmiterm([4 1 1 P2], -1, 1);

lmiterm([5 1 1 r2],1,1);

lmiterm([5 1 1 rou_u2],-1,1);

lmiterm([6 1 1 r1],1,1);

lmiterm([6 1 1 rou_x1],-1,1);

lmiterm([7 1 1 V],1,-1,'s');

lmiterm([8 1 1 P2B],-1,1,'s');

lmisys=getlmis;

[tmin,xfeas]=feasp(lmisys);

PP1=dec2mat(lmisys,xfeas,P1);

PP2=dec2mat(lmisys,xfeas,P2);

PP2A=dec2mat(lmisys,xfeas,P2A);

PP2B=dec2mat(lmisys,xfeas,P2B);

Ad=PP2A/PP1;

Bd=PP2B/PP2;

UU1=dec2mat(lmisys,xfeas,U);

VV1=dec2mat(lmisys,xfeas,V);

aalpha1=dec2mat(lmisys,xfeas,alpha1);

aalpha=dec2mat(lmisys,xfeas,alpha);

rr1=dec2mat(lmisys,xfeas,r1);

rrou_x1=dec2mat(lmisys,xfeas,rou_x1);

rrou_x=rrou_x1/rr1;

rr2=dec2mat(lmisys,xfeas,r2);

rrou_u2=dec2mat(lmisys,xfeas,rou_u2);

rrou_u=rrou_u2/rr2;

Cd=UU1^(-1)*VV1
